# Mechanistic Elucidation and Establishment of Drying Kinetic Models of Differential Metabolite Regulation in *Rheum palmatum* During Natural Sun Drying: An Integrated Physiology, Untargeted Metabolomics, and Enzymology Study

**DOI:** 10.3390/biology14080963

**Published:** 2025-08-01

**Authors:** Wen Luo, Jinrong Guo, Jia Zhou, Mingjun Yang, Yonggang Wang

**Affiliations:** 1 School of Life Science and Engineering, Lanzhou University of Technology, Lanzhou 730050, China; luowen@lut.edu.cn (W.L.); r15048521097@163.com (J.G.); yangmj@lut.edu.cn (M.Y.); 2 Gansu Food Inspection and Research Institute, Lanzhou 730199, China; henry_zona@126.com

**Keywords:** rhubarb, sun drying, drying kinetic model, metabolomics, enzyme activity

## Abstract

Rhubarb, a medicinal herb in Gansu Province, China, undergoes significant quality changes during sun-drying. This study investigated color changes, drying kinetics, anthraquinone (AQ) content, metabolic profiles, and enzyme activity during the process. Results showed that drying induced enzymatic browning, with the browning index (BI) progressively increasing over extended drying periods (4–16 h) and with greater slice thickness (2–8 mm). Catalase (CAT) activity first decreased and then increased, while polyphenol oxidase (PPO) activity decreased throughout drying. Slice thickness significantly affected AQ content, with the highest in 2 mm slices and the lowest in 4 mm slices. The drying process followed a logarithmic model (R^2^ = 0.99418, RMSE = 0.02310, and χ^2^ = 0.0005). Metabolomics analysis identified 631 differential metabolites, with 8 key metabolites linked to flavonoid biosynthesis, phenylalanine biosynthesis, and tyrosine metabolism. Fifteen enzymes were involved in metabolite synthesis and decomposition, though some enzyme activity trends contradicted metabolite changes. This study provides insight into rhubarb drying mechanisms and a basis for optimizing the drying process.

## Simple Summary

Rhubarb, a traditional medicinal herb in Gansu, China, undergoes critical quality changes during sun-drying, directly impacting the retention of bioactive components (e.g., anthraquinones) and visual characteristics. To optimize the drying process, this study investigated how slice thickness (2–8 mm) and drying duration affect color variation, enzyme activity, and medicinal efficacy. Results demonstrated significant enzymatic browning during the initial drying phase (4–16 h), with catalase activity continuously declining and polyphenol oxidase activity initially decreasing before rising, synchronizing with browning peaks. Thinner slices (2 mm) retained the highest anthraquinone content, while thicker slices (4 mm) exhibited the greatest loss. Moisture reduction followed a logarithmic model with high precision (R^2^ = 0.994). Metabolomic analysis identified 631 differential metabolites, including 8 key compounds linked to flavonoid biosynthesis, regulated by 15 enzymes, though some enzymatic trends contradicted metabolite dynamics. Enzymatic browning dominated quality degradation, with slice thickness critically influencing component retention. The complexity of the metabolic regulatory network was dynamically associated with enzyme activity. Optimizing slice thickness (2 mm) and controlling early drying (<16 h) effectively enhanced product quality, offering a transferable strategy for processing other medicinal herbs to minimize bioactive loss and improve market competitiveness.

## 1. Introduction

Rheum Radix et Rhizoma is a perennial herb belonging to the genus Rheum in the family Polygonaceae, including *Rheum palmatum* L., *Rheum tanguticum* Maxim.ex Balf, and Rheum officinale Baill [1]. The medicinal part of rhubarb is mainly its rhizome [2], which is classified as a top medicinal plant and has been used medicinally for more than 2000 years since it was recorded in the Divine Farmer’s Materia Medic [3]. Furthermore, its abundance of naturally occurring active constituents endow it with a diverse range of pharmacological effects. Rhubarb contains a variety of bioactive constituents such as polyphenols, anthraquinones (AQs), and flavonoids [4]. Particularly, AQs and flavonoids are the main active components in rhubarb [5], and more than 30 anthraquinones have been identified, including rhodopsin, aloe rhodopsin, and rhodopsin methyl ether [6], which have a variety of pharmacological activities, such as anticancer, anti-ischemic, anti-inflammatory, antiviral, antidiabetic, and so on [7]. Additionally, they are also used in traditional Chinese medicine (TCM) to clear heat and fire, cool blood and remove blood stasis, clear collaterals and provide detoxification, and for diarrhea [8].

In the field of Chinese medicine, decoction is used as the traditional preparation method for herbal compounding, while decoction pieces are another mainstream form of herbal medicine application [9]. Drying is a major part of the processing of decoction pieces, effectively removing excess water from herbs to prolong their storage time and facilitate transport [10], as well as limiting enzyme degradation and microbial growth and preserving their beneficial properties [11]. Especially for fresh rhubarb rhizomes, natural drying is difficult due to its coarseness and high moisture content, and it is prone to chaffing, mold, deterioration, and discoloration during processing. The 2020 edition of the *Pharmacopoeia of the People’s Republic of China* (hereinafter referred to as the Chinese Pharmacopoeia (ChP)) [12] stipulates that rhubarb should be cut into petals or segments and dried in bunches on strings or directly. The quality of herbs is significantly influenced by factors such as temperature, humidity, and airflow during the drying process [13]. Inappropriate drying techniques can result in the loss of active pharmaceutical ingredients and consequently affect the efficacy of the decoction pieces.

To date, the development of drying technology has formed two major categories: traditional and modern [10]. Traditional drying methods mainly include sun drying, shade drying, smoke drying, and hot air drying, while modern techniques have introduced a variety of combined drying technologies such as hot air drying, vacuum drying, microwave drying, freeze drying, and far-infrared drying [14]. Currently, the drying of genuine rhubarb still uses the more traditional smoke-drying method, which can retain a higher content of effective components. Sun et al. smoked rhubarb samples for more than two weeks and found that the pharmacological effects of smoke-dried rhubarb were significantly higher than those of other drying methods [11], but the long duration, poor product appearance, and environmental pollution issues cannot be ignored. In contrast, sun drying, as a green and low-carbon drying technology, is widely adopted for its low cost and the fact that it does not consume energy. However, the effects and quality of sun drying may be limited by factors such as weather, drying time, and the thickness of the medicinal materials. Therefore, it is necessary to consider these factors comprehensively to develop a reasonable drying process to ensure the quality and efficacy of rhubarb.

Drying is a complex process involving heat transfer and moisture migration [15]. Mastering the key control parameters of this process [16], such as temperature, slice thickness, and drying time, is crucial for improving drying efficiency and ensuring the quality of rhubarb slices [17]. Various mathematical models have been proposed to describe the drying processes of products like apples [18] and dioscorea opposite [19]. This study employs a drying kinetics model to deeply investigate the moisture migration behavior of rhubarb during the sun-drying process, aiming to optimize its drying process. Metabolomics is a scientific method that studies the composition of small molecular metabolites in biological systems, capable of revealing changes in metabolites comprehensively [20]. Yu et al., based on non-targeted metabolomics, successfully revealed the dynamic changes of 216 non-volatile compounds during the processing of round green tea [21]. Similarly, significant chemical composition changes occur in rhubarb during the sun-drying process, directly affecting its quality. Currently, there is relatively little metabolomic research on the sun-drying process of rhubarb. There is especially a lack of systematic analysis of the metabolite changes in rhubarb during the sun-drying process using non-targeted metabolomics techniques [22]. Therefore, this study explores the effects of slice thickness and drying time on the morphology, medicinal properties, drying kinetics characteristics, and metabolite changes in rhubarb slices to elucidate the mechanisms of the rhubarb sun-drying process, aiming to provide a scientific basis for rhubarb preparation and offering practical suggestions for optimizing the process.

## 2. Materials and Methods

### 2.1. Test Rhubarb

The fresh rhubarb used in this study was obtained from the standardized rhubarb cultivation base of Lixian Spring Pharmaceutical Co. Ltd., situated in Shangdu Village, Baihe Town, Lixian County, Longnan City, China (34°91′ N, 104°88′ E). The samples were selected from *Rheum palmatum* L. three years after transplanting and were identified as fresh roots of *Rheum palmatum* L. of the Polygonaceae family by Professor Yang Lin of the College of Life Science and Engineering, Lanzhou University of Technology. They were harvested in mid-March 2024, after the above-ground part of the plant had withered and the soil had thawed. The growing area has a temperate continental monsoon climate, with an altitude of about 2144 m above sea level, an average annual temperature of 8–11 °C, an average annual rainfall of about 600 mm, and a frost-free period of about 120 days.

### 2.2. Experimental Design

In this experiment, rhubarb of uniform diameter and moderate size was selected, the soil on the surface of freshly dug rhubarb was removed and washed, and the lateral roots (called ‘water roots’ by the local growers in Lixian County) were removed to leave the main root part. After the main root part was cleaned, a clean towel was used to wipe off the surface water and the test rhubarb was placed in a ventilated place to dry the excess water on the surface. Rhubarb slices with thicknesses of 2, 4, 6, and 8 mm (200 g per slice, 3 biological replicates per thickness; all experiments used *n* = 3 unless otherwise specified) were sun-dried under daytime temperatures of 14–27 °C and solar radiation of 800–1200 W/m^2^. Weight and water activity (a_w_) were measured every 2 h, while color parameters were monitored synchronously during the first 16 h (8 time points in total). This sampling protocol was adapted from previous studies on melon drying [23], with adjustments to accommodate rhubarb’s slower moisture migration caused by its fibrous structure: the sampling interval was extended from 90 min to 2 h. This approach effectively captures key changes during the rapid moisture evaporation stage in the early drying phase, accurately records the transitional stage as drying stabilizes in the mid-to-late phases, and improves experimental efficiency while ensuring data integrity. The drying process was terminated after 48 h when consecutive dry-weight differences fell below 1%, indicating equilibrium. All data were analyzed as the mean of three technical replicates. Weight, color parameters, and a_w_ were recorded as raw measurements from intermediate drying states without normalization.

### 2.3. Determination of Color Difference

To systematically investigate the color change dynamics of rhubarb during the drying process, this experiment employed a colorimeter to dynamically monitor the surface color of samples under sun-drying conditions. Using Cle Lab color parameters L*, a*, and b* on the surface of the dried material, a color change test for fresh material color and dry product material color before and after the change in the difference in the evaluation of the experimental process was undertaken. As such, we recorded the rhubarb color parameters (L*, a*, and b*) at each weighing time point during the drying process following ASTM E308-99 [24] Standard Practice for Computing the Colors of Objects by Using the CIE System. The browning index (BI) was calculated using the Formula (1):

(1) $$BI=\frac{100\times {(a}^{*}+1.75L^{*})}{5.645L^{*}+a^{*}-3.012b^{*}}$$

The color of rhubarb was evaluated using the CIE Lab color system, where L* (lightness index, range: 0–100) reflects surface brightness (higher values indicate whiter/brighter samples, lower values darker/blacker appearance), a* (red-green chromaticity, range: −128 to +127) represents red tones at positive values and green tones at negative values, and b* (yellow-blue chromaticity, range: −128 to +127) denotes yellow hues at positive values and blue hues at negative values. Measurements were conducted using a 3nh-YS3060 intelligent spectrophotometric colorimeter (Shenzhen ThreeNH Technology Co., Ltd., Shenzhen, China).

### 2.4. Preparation of Paraffin Sections

Sun-dried rhubarb slices (2 mm, 4 mm, 6 mm, and 8 mm thick) were cut into small pieces (≤10 mm × 10 mm) and immersed in 20% glycerol (Shanghai Zhongqin Chemical Reagent Co., Ltd., Shanghai, China) solution for 24 h to soften tissues, followed by vacuum infiltration for 2 h. The softened samples were then transferred to FAA fixative (glacial acetic acid (Sinopharm Chemical Reagent Co., Ltd., Shanghai, China) and absolute ethanol (Tianjin Baishi Chemical Co., Ltd., Tianjin, China) = 1:3) and fixed at 4 °C for 24 h. After fixation, the samples were sequentially washed and dehydrated through a graded ethanol series (50%, 70%, 85%, 95%, and absolute ethanol), with each step lasting 1 h. The dehydrated tissues were cleared twice in xylene (Sinopharm Chemical Reagent Co., Ltd., Shanghai, China) (1 h each) and infiltrated with paraffin wax (Leica Biosystems, Richmond, IL, USA) at 56–58 °C through three cycles (2 h each) to ensure complete penetration. Finally, the paraffin-embedded tissue blocks were mounted on a microtome holder, aligned perpendicular to the tissue plane, and sectioned at 5 μm thickness for subsequent microscopic observation [25]. A Nikon Eclipse 801 (Nikon Corporation, Tokyo, Japan) microscope with a total magnification of 40× was used.

### 2.5. Determination of Anthraquinone Content

Determination was carried out by high-performance liquid chromatography (HPLC) with reference to the General Technical Requirement of Part IV of the *Chinese Pharmacopoeia*, 2020 edition. The control solution and the test solution were pipetted into the sample vial of a high-performance liquid chromatograph (SPD-20, Shimadzu, Japan) at 10 μL. The sample was injected into the sample vial with automatic injection. The chromatographic conditions were as follows: the chromatographic column was Symmetry C18 (4.6 mm × 250 mm, 5 μm, Waters, Milford, MA, USA); the mobile phase was methanol (Chromatography Grade, Merck KGaA, Darmstadt, Germany) and 0.1% phosphoric acid (Chromatography Grade, Sinopharm Chemical Reagent Co., Ltd., Shanghai, China) in water (85:15); the flow rate was 1 mL/min; the column temperature was 30 °C; and the detection wavelength was 254 nm. The peak area of aloe vera rhododendron, rhodizontic acid, rhododendron, rhododendron phenol, rhododendron methyl ether, and rhododendron methyl ether were then obtained by recording the peak area of the sample. The anthraquinone content in the test material (expressed on a dry-weight basis (DW)) was calculated by the external standard method according to Formula (2):

(2) $$C_{x}=C_{R}\frac{A_{x}}{A_{R}}$$

where C_x_ is the concentration of the test sample; A_x_ is the peak area or peak height of the test sample; C_R_ is the concentration of the control substance; and A_R_ is the peak area or peak height of the control.

### 2.6. Determination of Drying Kinetic Parameters Including Dry Basis Moisture Content (Mt), Moisture Ratio (MR), Drying Rate (DR), and Water Activity (a_w_)

During the drying process, the M_t_ of rhubarb was calculated according to Equation (3):

M_t_ = (m_t_ − m_s_)/m_s_ (3)

where m_t_ is the total sample mass at time t (wet weight, g) and m_s_ is the absolute dry matter mass determined by the constant weight method. Samples were dried in an oven at 105 °C until reaching constant weight (±0.1 g) (unit: g).

And then, MR was calculated according to Equation (4):

MR = (M_t_ − M_e_)/(M_0_ − M_e_) (4)

where M_e_ is the equilibrium M_t_ of rhubarb and generally in the actual experimental process the default value is the M_t_ at the end of the drying test, and M_0_ is the initial M_t_. This is because M_e_ is much smaller than M_t_ and M_0_ and so in the actual calculation process M_e_ is often ignored; consequently, the moisture ratio can be simplified from Equation (4) to Equation (5):

MR = M_t_/M_0_ (5)

Meanwhile, DR was calculated according to Equation (6):

DR = (M_t_ − M_t+Δt_)/Δt (6)

where M_t+Δt_ is the M_t_ at the moment t + Δt and Δt is the drying time between two adjacent drying times (unit: s).

a_w_ indicates the amount of water in the sample, with a value between 0 (completely dry) and 1 (pure water). Measurements were made using a HD-3A benchtop water activity meter (Huake Instruments Co., Ltd., Wuxi, China). Samples were ground before analysis and each sample was analyzed in triplicate.

### 2.7. Metabolomics Analysis

Based on the systematic analysis of rhubarb’s morphological and medicinal properties in this study, 4 mm-thick slices exhibited optimal color stability and textural integrity during sun-drying. Although the 4 mm-thick slices exhibit relatively low anthraquinone content (20.80 mg/g) they still hold potential for subsequent metabolomics research. To investigate the metabolic regulatory mechanisms under suboptimal drying thickness, an independent batch of 4 mm-thick rhubarb slices (200 g, *n* = 6) was prepared and sun-dried under identical environmental conditions. From the onset of drying, 20 g samples were randomly collected every 120 min, immediately flash-frozen in liquid nitrogen (local supplier), and stored at −80 °C in an ultra-low temperature freezer for subsequent processing. Samples collected at 4 h, 6 h, 8 h, 12 h, and 16 h time points were ultimately selected for untargeted metabolomics analysis.

#### 2.7.1. Sample Processing Methods Required for Metabolomics

The test rhubarb was placed in a mortar and ground into powder. A total of 50 mg of the sample was weighed and 600 μL of 70% methanol (Merck KGaA, Darmstadt, Germany) was added. It was vortexed 6 times, each time for 30 s, and subsequently placed in −20 °C environment for 30 min. It was then centrifuged at 12,000 rpm for 3 min, after which the supernatant was taken, the samples were filtered by microporous filter membrane (0.22 μm organic system, Beijing Lanjieke Technology Co., Ltd., Beijing, China) and then stored in the injection bottle for UPLC-MS/MS analysis.

#### 2.7.2. Mass Chromatography Acquisition Conditions

Ultra-performance liquid chromatography-mass spectrometry (UPLC-MS/MS) analysis was performed using an LC-30A UPLC system (Shimadzu, Kyoto, Japan) coupled with a TripleTOF 6600+ mass spectrometer (SCIEX, Shanghai, China) to analyze the metabolites in rhubarb samples with different drying times. An ACQUITY UPLC HSS T3 column (1.8 μm, 2.1 mm × 100 mm, Waters Corporation, Milford, MA, USA) was used, and the column temperature was maintained at 40 °C. The flow rate was 0.4 mL/min and the injection volume was 4 μL. The mobile phase A was ultrapure water (0.1% formic acid (Merck KGaA, Darmstadt, Germany)) and the mobile phase B was acetonitrile (Merck KGaA, Darmstadt, Germany) (0.1% formic acid). The mass spectrometry analysis was performed in multiple reaction monitoring (MRM) mode, using the positive and negative ion modes of the electrospray ionization source (ESI). The settings were as follows: ion source temperature at 550 °C, ion spray voltage at 5000 V in positive ion mode and −4000 V in negative ion mode, and the ion source gas pressure was maintained at 50 psi for both modes.

#### 2.7.3. Differential Metabolite Analysis

For the metabolite analysis of sun-dried rhubarb, differential metabolite screening was performed using the MetaboAnalyst online platform, with a *p*-value of 0.05, a VIP value of 1, and a differential fold change (FC) screening threshold of 2 to ensure that biologically significant metabolites were screened. The screened differential metabolites were then subjected to pathway analysis using the Kyoto Encyclopedia of Genes and Genomes (KEGG) database, which provides a wealth of pathway information essential for understanding the role of these differential metabolites in biological processes and their interrelationships. To further differentiate the metabolite expression differences between different groups of samples, we used principal component analysis (PCA) and orthogonal partial least squares discriminant analysis (OPLS-DA) to distinguish the metabolite expression differences between different groups of samples. The results of these analyses not only help to understand the metabolic properties of sun-dried rhubarb but also provide important information for further biological studies.

### 2.8. Enzyme Activity Assay

Accurately weigh 1.0 g of rhubarb samples dried for 4 h, 6 h, 8 h, 12 h, and 16 h, add a small amount of 0.1 M phosphate-buffered saline (PBS, Beijing Lanjieke Technology Co., Ltd., Beijing, China), and homogenize under ice-bath conditions. Transfer the homogenate to a 10 mL graduated test tube, adjust the volume to the mark with PBS, and centrifuge at 12,000× *g* for 15 min at 4 °C. The resulting supernatant is the crude enzyme extract. Enzyme activities were expressed on a dry-weight basis (DW).

#### 2.8.1. Catalase (CAT)

CAT [26] was measured using the catalase activity test kit (Solarbio, BC0200, Beijing Solarbio Science & Technology Co., Ltd., Beijing, China), the specific steps are as follows: preheat the enzyme labeler for 30 min, adjust the wavelength to 240 nm, and zero the wavelength with distilled water; configure the working solution of CAT assay; add 25 mL of reagent II to 25 mL of reagent I in the kit and mix it well to use it as the working solution; put the working solution of CAT assay in a 25 °C place; add 10 μL of sample extract and 190 μL of working solution into the 96-well plate, mix and time immediately, record the initial absorbance value A1 at 240 nm and the absorbance value A2 after 1 min, and calculate ΔA = A1 − A2. repeat three times.

#### 2.8.2. Polyphenol Oxidase (PPO)

The PPO [26] was measured using a polyphenol oxidase activity kit (Solarbio, BC0195, Beijing Solarbio Science & Technology Co., Ltd., Beijing, China) with the following steps: preheat the enzyme marker for 30 min, adjust the wavelength to 525 nm, and zero the wavelength with distilled water; add the assay sample and the control sample in the EP tube, respectively, and add 180 μL of the sample to be tested in the tube to be tested, along with 720 μL of reagent I and 180 μL of reagent II; in the control tube, add 180 μL of boiled (95 °C water bath for 5 min) sample, 720 μL of reagent I, and 180 μL of distilled water; after, put in a water bath at 25 °C for 10 min and a water bath at 95 °C for 5 min, mix thoroughly, and centrifuge at 25 °C for 10 min; and then, take the supernatant and detect the absorbance of the assay tube and the control tube at the wavelength of 525 nm. Repeat three times.

#### 2.8.3. Key Enzymes in Metabolic Pathways

The activities of key enzymes involved in the synthesis and degradation of differential metabolites (15 enzymes including chalcone isomerase (CHI), anthocyanidin reductase (ANR), flavonoid 3′-monooxygenase (TT7), flavanone 3-hydroxylase (F3H), flavanoid 3′,5′-hydroxylase (CYP75A), 4-hydroxyphenylpyruvate dioxygenase (PDS1), maleylpyruvate isomerase (nagL), homogentisate 1,2-dioxygenase (HGO), acylpyruvate hydrolase (FAHD1), 3-fumarylpyruvate hydrolase (nagK), caffeate O-methyltransferase (OMT1), coniferyl-aldehyde dehydrogenase (ALDH2C4), coniferyl-alcohol glucosyltransferase (UGT72E), sinapate 1-glucosyltransferase (UGT84A2), and 4-coumarate—CoA ligase (4CL1)) were determined using ELISA kits (Shanghai Tongwei Biotechnology Co., Ltd., Shanghai, China).

The specific procedures are as follows: First, a standard curve was constructed by plotting gradient concentrations of standards (μg/mL) on the *x*-axis against corresponding absorbance values (ODs) on the *y*-axis. A linear regression equation (*y* = *ax* + *b*) was fitted using the least squares method, with the coefficient of determination (R^2^) required to exceed 0.99. Next, the measured ODs of test samples were substituted into the equation to calculate actual concentrations, which were then corrected by multiplying with the dilution factor applied during sample pretreatment (e.g., multiplied by 5 for a 1:5 dilution). The enzyme activity was thereby determined. According to the International Union of Biochemistry and Molecular Biology (IUBMB) standards, one unit of enzyme activity (U) is defined as the amount of enzyme required to catalyze the conversion of 1 μmol of substrate per minute under standardized reaction conditions (25 °C, pH 7.4). Final results are expressed as enzyme activity per unit volume (U/L).

### 2.9. Statistical Analysis

Excel 2020 software was used for statistical and preliminary processing of the data, Origin Pro2021 software was used for nonlinear curve fitting analysis of the model, and IBM SPSS-25 software was used for linear regression analysis and analysis of variance.

## 3. Results

### 3.1. Macroscopic Analysis of Dried Rhubarb

**Figure 1:**
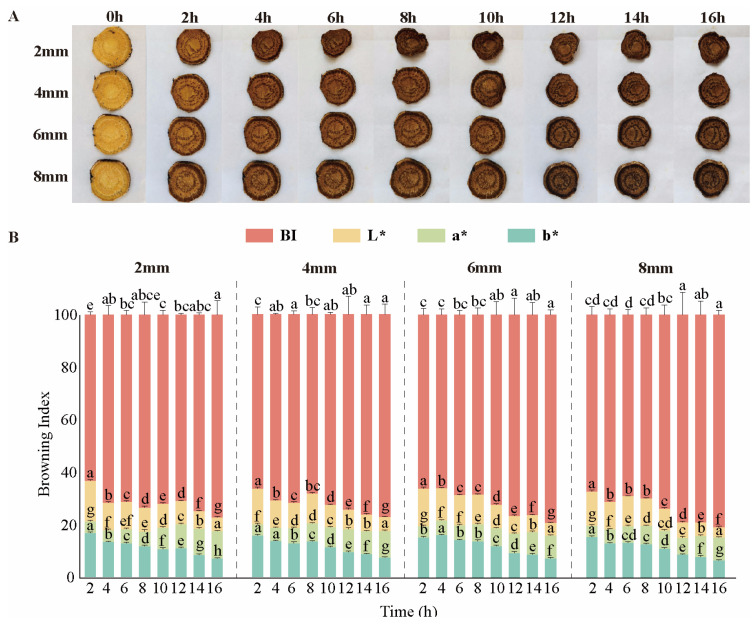
(**A**) Schematic diagram of the drying process of fresh rhubarb with different slice thicknesses; (**B**) variation in the browning index (BI) with drying time for sun-dried rhubarb at different slice thicknesses. Different lowercase letters (a–h) indicate significant differences among groups (*p* < 0.05), while shared letters denote no significant difference. The same applies below.

During the drying process, rhubarb exhibits a distinct browning phenomenon, and the slice thickness (2 mm, 4 mm, 6 mm, and 8 mm) has no significant effect on the color change in rhubarb (Figure 1A). As the drying time extends, the BI exhibited an increasing trend. (Figure 1B). Compared to fresh rhubarb, dried rhubarb L* and b* decreased while a* increased, and its color darkened, transitioning from yellowish brown to reddish brown with uneven surface color, dense wrinkles, and a texture that is harder than fresh rhubarb. The cross-section has a distinct brocade pattern and brown punctate secretory cavities, with a clear oily luster visible; it lacks a fresh fragrance, the bitterness is somewhat reduced, and it does not exhibit the astringent taste characteristic of fresh rhubarb. From a medicinal evaluation perspective, the sun-dried rhubarb slices meet the “morphological evaluation” criteria for rhubarb drying methods in the 1963 edition of the ChP, which states “the best quality is solid in texture, with a distinct brocade pattern on the cross-section, reddish-brown color, oily, fragrant, bitter but not astringent, and sticky when chewed”. Subsequent editions of the ChP have not further detailed the specific evaluation requirements for rhubarb’s characteristics, and sun-dried rhubarb slices still meet these basic requirements, thus maintaining a broad applicability in the field of medicine.

### 3.2. Microscopic Analysis of Dried Rhubarb

**Figure 2:**
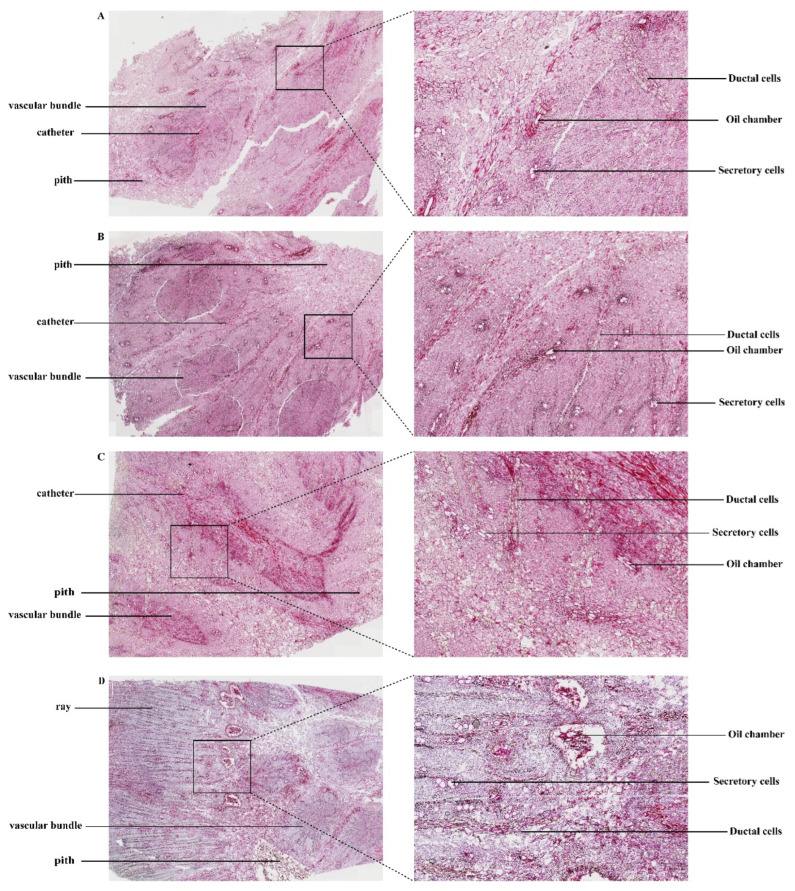
Cross-sectional microstructure and local enlargement of rhubarb at different slice thicknesses. (**A**) Cross-sectional microstructure and local enlargement of rhubarb with a slice thickness of 2 mm. (**B**) Cross-sectional microstructure and local enlargement of rhubarb with a slice thickness of 4 mm. (**C**) Cross-sectional microstructure and local enlargement of rhubarb with a slice thickness of 6 mm. (**D**) Cross-sectional microstructure and local enlargement of rhubarb with a slice thickness of 8 mm.

Paraffin sectioning of the dried rhubarb rhizome was performed, and the cross-sectional microscopic structure is shown in Figure 2. The cross-section, from the exterior to the interior, consists of the cork layer, cortex, phloem, cambium, and xylem. The phloem (occupying over 50% of the root) contains numerous annularly arranged oil chambers (larger in the outer region and smaller inward), surrounded by 11–13 secretory cells. Thin sections clearly reveal volatile oil droplets in the secretory cells, while thick sections demonstrate three-dimensional connectivity between oil chambers, indicating that their gradient structure effectively traps volatile oils. Additionally, starch granules in phloem parenchyma cells reduce cell collapse through water retention, minimizing component loss. The xylem contains medullary rays (composed of five columns of xylem parenchyma cells), which radiate to connect the cambium and phloem rays. Thin sections may disrupt the medullary rays, obscuring polysaccharide migration pathways, whereas thick sections preserve their continuity, confirming their directional transport function—driving hydrophilic components (e.g., polysaccharides, proteins) to accumulate in the phloem and transferring xylem volatile oils to phloem oil chambers to prevent oxidation. The cork layer (4–11 cell layers) exhibits a compact barrier structure in thin sections, while thick sections reveal its tendency to detach at the junction with the cortex, suggesting that drying speed must match structural tolerance; slow drying maintains barrier integrity, reducing volatile loss, while rapid drying still allows phloem oil chambers to stabilize components. The cambium, composed of small cells, activates phenolic and flavonoid synthesis under dehydration stress and stimulates adjacent cells to secrete mucilage to stabilize oil chamber structures. The proportion of the phloem and the width of the medullary rays create favorable conditions for storing volatile oils, proteins, polysaccharides, and other compounds, which are critical indicators of rhubarb’s medicinal value.

### 3.3. Changes in CAT and PPO Activities During the Drying Process of Rhubarb

**Figure 3:**
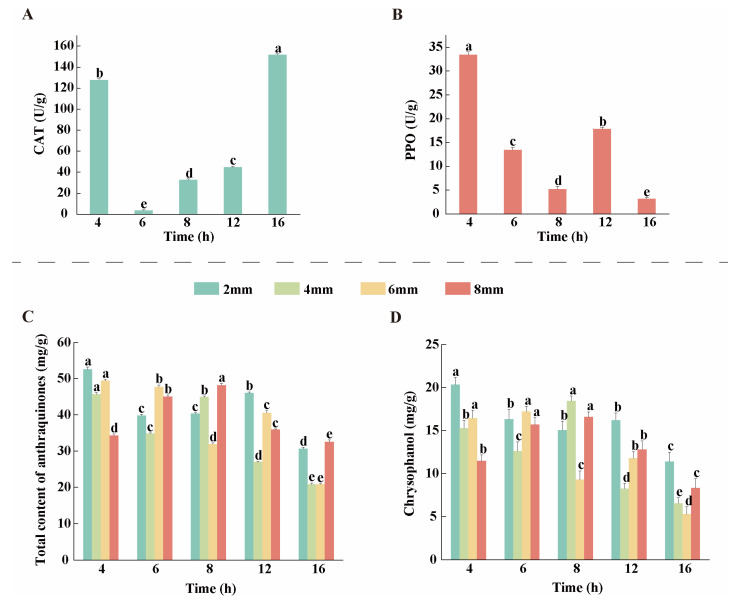
(**A**) Trends of CAT. (**B**) Trends of PPO. (**C**) Content of total anthraquinone compounds. (**D**) Chrysophanol content. Different lowercase letters indicate significant differences at the *p* < 0.05 level. All data are expressed on a dry-weight basis (DW).

CAT is one of the key protective enzymes widely present in plant tissues, and the changes in its activity are closely related to plant resistance [27]. As shown in Figure 3A, CAT activity increased significantly to 127.60 U/g at 4 h during the initial stage of drying, then it decreased to the lowest point of 3.41 U/g at 6 h. During the final stage of drying (16 h), CAT activity increased again and reached a peak value of 151.66 U/g. The overall trend of PPO activity showed a decreasing trend (Figure 3B) at the initial stage of drying. The PPO activity reached its highest point of 33.43 U/g at 4 h and then gradually decreased, until at 12 h of drying the PPO activity rebounded to 17.85 U/g; however, at the final stage of drying, the PPO activity again decreased and finally fell to 3.16 U/g at 16 h.

### 3.4. Analysis of AQ Content

During the drying process, the content of AQ in rhubarb decoction pieces with different slice thicknesses (2 mm, 4 mm, 6 mm, and 8 mm) and drying times (4 h, 6 h, 8 h, 12 h, and 16 h) showed significant changes. According to the 2020 edition of the ChP, rhubarb contains not less than 1.5% of total anthraquinone (TAQ) in terms of the total amount of rhubarb rhodopsin, rhubarbic acid, rhodopsin, rhubarb phenol, and rhubarbine methyl ether.

As shown in Figure 3C, the TAQ content of rhubarb slices with different thicknesses exhibited significant differences during the drying process. The 2 mm slices showed the highest initial TAQ content (52.58 mg/g at 4 h), which decreased to 30.59 mg/g by the end of drying (16 h, a 41.8% reduction) yet remained consistently higher than other groups. The 4 mm slices displayed the lowest overall TAQ content, decreasing from 45.70 mg/g (4 h) to 20.80 mg/g (16 h, a 54.5% reduction), with values converging closely to those of the 6 mm slices at 16 h. The 6 mm slices experienced the most severe degradation, with TAQ content dropping by 57.9% from 4 h to 16 h. The 8 mm slices showed a unique trend as TAQ content rose to a peak during 4–8 h but declined to 32.46 mg/g by 16 h, slightly below the initial level. Overall, although TAQ content generally decreased with prolonged drying time, it remained compliant with pharmacopeial standards, and its dynamic variation patterns were jointly governed by slice thickness and drying duration. The total content of anthraquinones in rhubarb can serve as a direct indicator of its medicinal quality. Among the five anthraquinones, chrysophanol exhibits the highest content (Figure 3D). As a key component responsible for the purgative and laxative effects as well as the antibacterial and anti-inflammatory activities of rhubarb, its content directly determines the overall therapeutic potency of the medicinal material [28]. To maximize the retention of total anthraquinones, drying time should be adjusted according to slice thickness, thereby ensuring the quality and therapeutic efficacy of rhubarb medicinal materials.

### 3.5. Drying Model and Data Fitting Analysis

#### 3.5.1. Effect of Different Slice Thickness on the Drying Rate (DR) of Rhubarb

**Figure 4:**
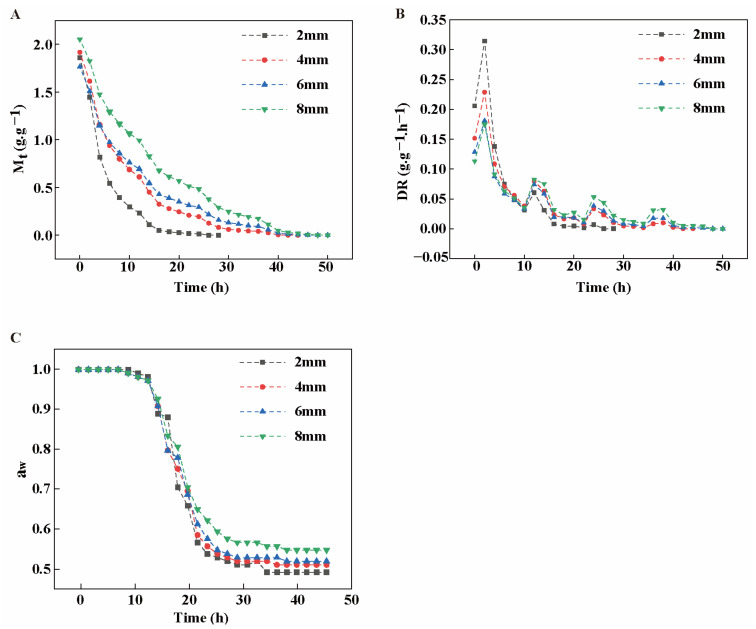
(**A**) Effect of different slice thickness on M_t_; (**B**) effect of different slice thickness on DR; (**C**) effect of different slice thickness on a_w_.

Figure 4A reveals the significant effects of slice thicknesses (2 mm, 4 mm, 6 mm, and 8 mm) on the dry basis moisture content (Mt) of rhubarb during the drying process. It can be observed that the Mts all showed a decreasing trend with time, and in the initial stage the decreasing rate was faster and then gradually leveled off. Differences existed between rhubarb thicknesses, with the 2 mm sample showing a faster decrease in dry basis moisture content at the initial stage of drying, while the 8 mm sample showed a slower decrease throughout the drying process. The drying rate peaked at the initial stage of drying followed by a rapid decrease (Figure 4B), with the sample with a thickness of 2 mm having the highest DR at the initial stage and the sample with a thickness of 8 mm having the lowest DR. This is consistent with the trend of Mt in Figure 4A, further verifying the effect of slice thickness on the drying process. At the end of the drying process, rhubarb of different slice thicknesses showed aw values (Figure 4C) that were below the microbial safety limit of 0.6, proving that it is safe in terms of microbial hazards [29].

#### 3.5.2. Comparison of Kinetic Models for Drying Rhubarb with Different Slice Thicknesses

In this study, four classical models (Table S1), including Lewis, Wang and Singh, Weibull, and logarithmic, were used to nonlinearly fit the regression analysis of the moisture ratio (MR) of different slice thicknesses during rhubarb drying. These models are effectively applicable to optimizing the drying kinetics processes in agricultural products such as garlic slices [30] and mushrooms [31]. Rhubarb sections are porous and heterogeneous tissues with similar drying behavior to the above plant materials. The goodness of fit of the mathematical models was indicated by the coefficient of determination (R^2^), residual squared (RMSE), and Chi-square validation value (χ^2^); the closer R^2^ is to 1, and the closer RMSE and χ^2^ is to 0 the better the fit [32]. The statistical analysis of R^2^, χ^2^, and RMSE of these four models are shown in Table 1, which shows that the Lewis, Wang and Singh, and logarithmic models have a high degree of fit, with the logarithmic model having the best fit with an R^2^ of 0.99418, and an RMSE and χ^2^ of 0.02310 and 0.00055, respectively, which were the most suitable for predicting and analyzing the change in moisture ratio in the rhubarb drying process among the four models selected in the experiment. Therefore, the logarithmic model was chosen as the kinetic model for rhubarb drying studies, and its fitting equation was MR = a exp(−kt) + c.

#### 3.5.3. Logarithmic Model Simulation of Rhubarb with Different Slice Thicknesses

Based on the fitting of the logarithmic data model for the test data of rhubarb with different slice thicknesses, the values of drying coefficients and evaluation indexes were obtained, as shown in Table 2. The parameter a represents the MR of rhubarb at the commencement of the drying process, whereas the parameter k correlates with the drying rate, indicating the rapidity of the decrease in moisture content. As evidenced in Table 2, an increase in slice thickness did not result in a notable alteration to parameter a; however, it did lead to a substantial decline in parameter k. This suggests that the drying rate of rhubarb diminished as the thickness of the slices augmented. This phenomenon may be attributed to the fact that thicker slices require a longer time to remove the internal moisture, thus slowing down the overall DR [33].

#### 3.5.4. Logarithmic Model Validation

**Figure 5:**
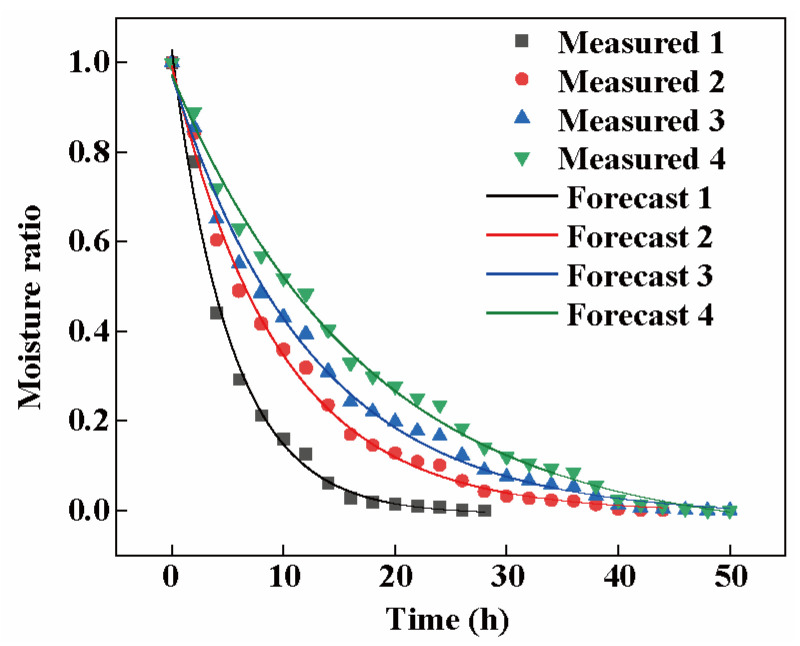
Comparison of predicted and experimental values of the constructed logarithmic model.

In order to further test the accuracy of the logarithmic model in predicting the drying process of rhubarb, four sets of data with different slice thicknesses in the experiment were compared with the logarithmic model MR. As can be seen from Figure 5, the measured values of the experimental process fit well with the predicted values of the model, with an R^2^ value of more than 0.99. This high degree of correlation suggests that the logarithmic model has extraordinary predictive ability for the drying process and is well-suited for describing and predicting the dynamic changes in the moisture within the rhubarb slices during the entire drying process.

### 3.6. Metabolomics Analysis of Sun-Dried Rhubarb Decoction Pieces

#### 3.6.1. Metabolite Identification Results and Analysis

**Figure 6:**
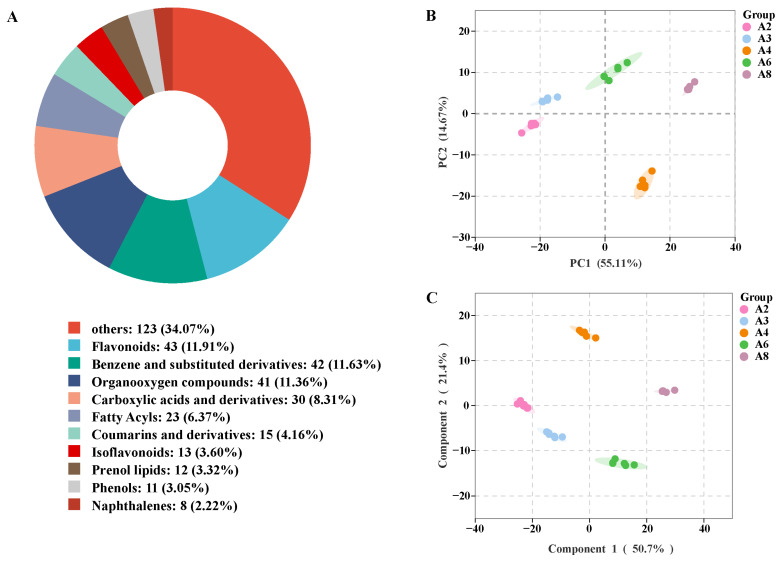
Metabolomics analysis. (**A**) Types of differential metabolites of rhubarb at different drying periods; (**B**) PCA of rhubarb metabolites; (**C**) OPLS-DA analysis of rhubarb metabolites.

A total of 631 differential metabolites were identified and screened in the sun-dried rhubarb samples using non-targeted metabolomics techniques, as illustrated in Figure 6A. When constructing a pie chart, a threshold of 2% was set for other categories, with any percentages below this value classified as “Other categories”. The identified metabolites were classified into the following categories: 43 Flavonoids, 42 Benzene and Substituted Derivatives, 41 Organooxygen Compounds, 30 Carboxylic Acids and Derivatives, 23 Fatty Acyls, 15 Coumarins and Derivatives, 13 Isoflavonoids, 12 Prenol lipids, 11 Phenols, 8 Naphthalenes, and other substances were identified. As a crucial medicinal plant, the quality of rhubarb is typically evaluated based on the content of its active components. Flavonoids and benzene-derived compounds (e.g., phenylpropanoids and anthraquinones) are key bioactive constituents in rhubarb, directly determining its medicinal value. Among these differential metabolites, flavonoids account for the largest proportion (11.91%), followed closely by benzene derivatives (11.63%). Flavonoids, characterized by their polyhydroxyl structures, confer robust antioxidant capacity to rhubarb, neutralizing free radicals to delay oxidative deterioration and stabilizing components by chelating metal ions to inhibit enzymatic browning [34]. Benzene derivatives, such as phenylpropanoids and anthraquinones, are directly linked to rhubarb’s core pharmacological effects. For instance, the ratio of free to conjugated anthraquinones governs the intensity of laxative activity, while phenolic acids derived from benzene rings enhance therapeutic efficacy through anti-inflammatory and antimicrobial actions. These two classes of compounds exhibit functional coupling at the metabolic level—flavonoids mitigate oxidative degradation of phenylpropanoids through antioxidant activity while the bioactivity of benzene derivatives relies on flavonoids to maintain structural stability [35]. This chemical–ecological interaction network forms the multidimensional indicator basis for rhubarb quality evaluation, integrating compositional balance and functional synergy.

Through principal component analysis (PCA) we explored the overall characteristics of rhubarb samples from five different drying times (DTs). Figure 6B shows the significant differences among rhubarb samples. The first principal component (PC 1) accounted for 55.11% of the total variance, while the second principal component (PC 2) accounted for 14.67%. This indicates that there were significant metabolic differences between the samples from the five DTs, but the differences between the 4 h and 6 h drying periods were relatively small. Orthogonal partial least squares discriminant analysis (OPLS-DA) score plots further revealed a clear separation between the samples from the five drying periods. This separation was based on the principles of *p*-value (<0.05) and VIP (≥1), thus confirming that the five DT samples had their own unique metabolic profiles, as shown in Figure 6C.

**Figure 7:**
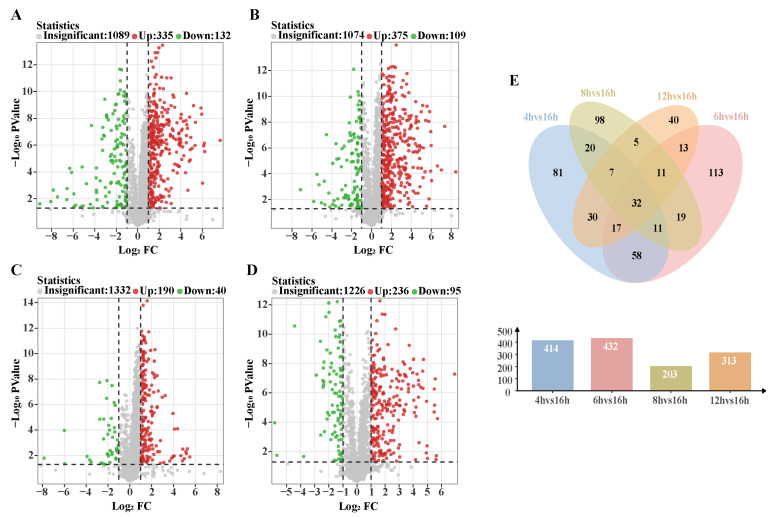
(**A**) Volcanic diagram of the expression of differential metabolites of rhubarb at 4 h and 16 h. (**B**) Volcanic diagram of differential metabolites expression of rhubarb at 6 h and 16 h. (**C**) Volcanic diagram of differential metabolites expression of rhubarb at 8 h and 16 h. (**D**) Volcanic plot of the expression of differential metabolites of rhubarb at 12 h and 16 h. (**E**) Venn diagram of differential metabolites of rhubarb at different drying times.

Setting the FC value threshold to 2 and the *p* value threshold to 0.05 generated volcano plots of the differential metabolites in each comparison group, which helped to quickly identify the differences in metabolite expression levels between the two samples and the statistical significance of these differences. Figure 7A–D shows that 447 (335 up-regulated, 112 down-regulated), 484 (375 up-regulated, 109 down-regulated), 230 (190 up-regulated, 40 down-regulated), and 331 (236 up-regulated, 95 down-regulated) metabolites were expressed significantly differently between the 16 h sun-drying period compared to the 4, 6, 8, and 12 h drying periods, respectively. Expression of the differential metabolites was up-regulated at 4, 6, 8, and 12 h of drying compared to 16 h of drying. A total of 32 differential metabolites were observed across the five DTs after taking the intersection of each comparison group in the Venn diagram (Figure 7E). Notably, each comparison group had its own unique differential metabolites.

#### 3.6.2. Analysis of Differential Metabolic Pathways

**Figure 8:**
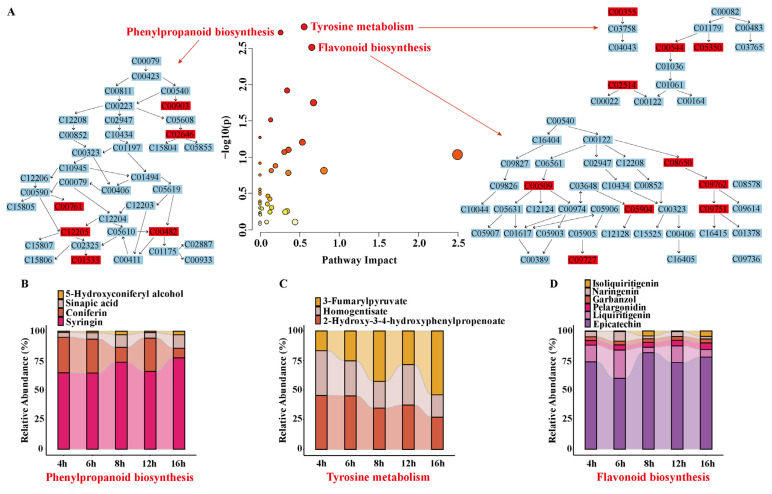
(**A**) Significant metabolic pathways. (**B**) Relative abundance of differential metabolites in the phenylpropanoid biosynthesis pathway. (**C**) Relative abundance of differential metabolites in the tyrosine metabolism pathway. (**D**) Relative abundance of differential metabolites in the flavonoid biosynthesis pathway.

Forty metabolic pathways of sun-dried rhubarb were screened by KEGG enrichment analysis of the screened differential metabolites, and it was found that the differential metabolites were significantly enriched in the pathways related to the synthesis of plant secondary metabolites. As shown in Figure 8A, the pathway analysis overview plot has the -log(*p*) value of enrichment analysis as the vertical axis and the effect value of topological analysis as the horizontal axis. Each bubble represents a metabolic pathway; the darker the bubble color, the lower the *p* value and the more significant the enrichment. A *p* value of <0.005 and an impact >0.1 were used as the screening criteria for significant metabolic pathways, and the following three significant metabolic pathways were obtained: tyrosine metabolism, phenylpropanoid biosynthesis, and flavonoid biosynthesis [36].

**Figure 9:**
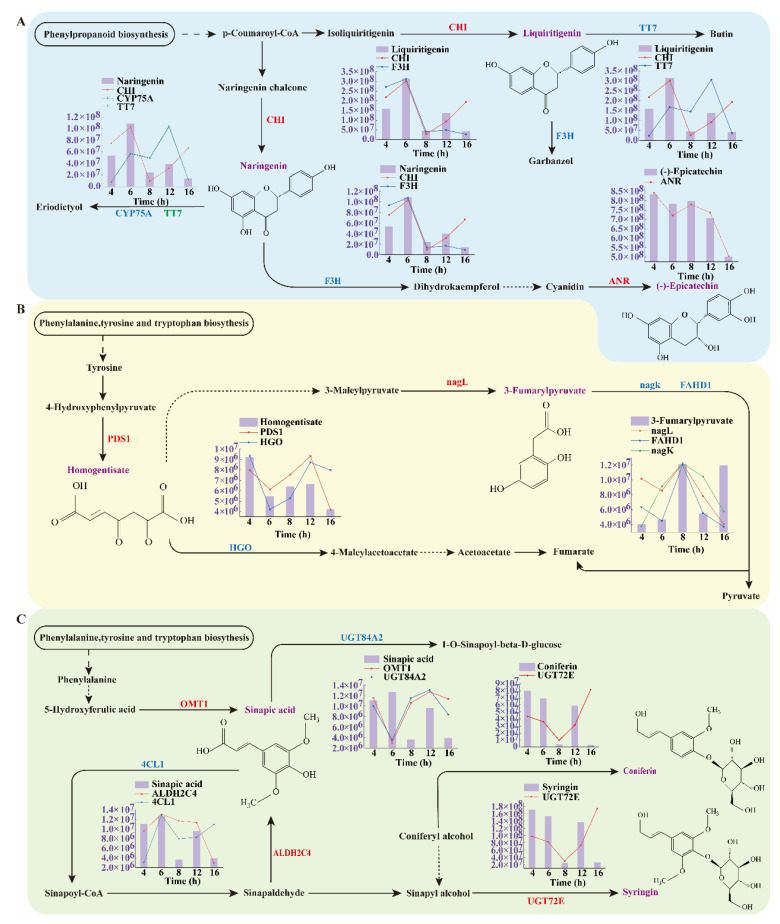
Biosynthetic pathways. (**A**) Flavonoid biosynthesis. (**B**) Tyrosine metabolism. (**C**) Phenylpropanoid biosynthesis.

The relative abundance of differential metabolites enriched by these three major metabolic pathways is demonstrated in Figure 8B–D. In this paper, special attention was paid to eight differential metabolites with significant changes, including Liquiritigenin, Naringenin, (-)-Epicatechin, Homogentisate, 3-Fumarylpyruvate, Sinapic acid, Coniferin, and Syringin, and we further explored their roles in metabolic pathways and their regulatory mechanisms. For a more comprehensive understanding of the metabolic changes in Rheum officinale during the drying process, the key metabolic pathways were mapped in detail in this paper (Figure 9), and the dynamic changes in the related metabolites and enzyme activities were visualized in the form of bar charts. These quantitative data provide an important basis for revealing the molecular mechanisms of metabolic regulation during the drying process of rhubarb.

#### 3.6.3. Changes in the Activities of Key Enzymes During Rhubarb Drying

In the flavonoid biosynthetic pathway (Figure 9A), chalcone isomerase (CHI) plays a key role and can promote the accumulation of flavonoids [37]. Liquiritigenin is a core intermediate whose synthesis is regulated by the enzyme CHI, which is converted from Isoliquiritigenin. The two enzymes, F3H and TT7, act synergistically in the metabolism of Liquiritigenin, catalyzing its conversion to Garbanzol and Butin, respectively, and the changes in the activities of the two enzymes are consistent with the trend of the changes in the content of Liquiritigenin, showing their synergistic roles in the same metabolic pathway. It is noteworthy that the activity change in CHI enzyme in the first four drying periods followed the trend of Liquiritigenin, while in the last period, when Liquiritigenin decreased, the activity of CHI enzyme showed an increasing trend. In this metabolic pathway, the production of Naringenin is also regulated by the CHI enzyme, which is converted from Narigenin chalcone. Naringenin is converted to Eriodictyol by the combined action of CYP75A and TT7. Another downstream branch of Naringenin is the formation of Dihydrokaempferol catalyzed by F3H. The trend of CHI enzyme regulation of Naringenin production was similar to that of Liquiritigeninde, with activity changes in the first four drying periods coinciding with Naringenin levels and CHI enzyme activity increasing during the final period when Naringenin was decreasing. In addition, the generation of (-)-Epicatechin is a result of the conversion of Cyanidin catalyzed by ANR enzyme, and the change in ANR enzyme activity coincided with the trend of (-)-Epicatechin. This suggests that ANR enzyme plays a key role in the generation of (-)-Epicatechin.

In the tyrosine metabolic pathway (Figure 9B), Homogentisate was generated by the conversion of 4-Hydroxyphenylpyruvate under the regulation of PDS1, and then was further converted to 4-Maleylacetoacetate by the catalysis of the downstream branch of the HGO enzyme; the changes in the activities of the two enzymes, PDS1 and HGO, showed at first a decrease and then an increase with the increase in drying time, which was highly consistent with the change trend of Homogentisate. In addition, Homogentisate is also involved in the generation of 3-Maleylpyruvate. The other differential metabolite in this metabolic pathway, 3-Fumarylpyruvate, is 3-Maleylpyruvate catalyzed by nagl, and the branch downstream of 3-Fumarylpyruvate by the FAHD1 and nagk together produce Pyruvate and Fumarate. All three enzymes showed partially inconsistent trends with the changes in 3-Fumarylpyruvate. nagl and FAHD1, which are similar enzymes, showed opposite activities to 3-Fumarylpyruvate at two drying times, 4 h and 16 h, whereas the nagk enzyme showed the opposite trend only at the drying time of 16 h.

In the phenylpropane biosynthetic pathway (Figure 9C), Sinapic acid, as a key metabolite in this metabolic pathway, is regulated by several enzymes. The synthesis of Sinapic acid starts from 5-Hydroxyferulic acid and is converted to Sinapic acid under the catalysis of the enzyme OMT1. The change in the activity of OMT1 is consistent with the trend of Sinapic acid in this process. Subsequently, Sinapic acid is converted to 1-O-Sinapoyl-beta-D-glucose by the action of UGT84A2 enzyme, and the change in UGT84A2 enzyme activity also matches the trend of Sinapic acid. Another branch of Sinapic acid metabolism begins with the 4CL1-catalyzed formation of Sinapoyl-CoA, which is further converted to Sinapaldehyde, which is a direct precursor metabolite of Sinapic acid and is converted to Sinapic acid catalyzed by the ALDH2C4 enzyme. The ALDH2C4 enzyme showed a consistent trend with Sinapic acid, showing at first a rise, then a fall, followed by a rise and then a fall; conversely, 4CL1 showed a consistent trend with the metabolite during the first four periods of drying, and showed the opposite trend only in the last period. Sinapic acid is also involved in the production of other metabolites, such as the conversion to Sinaply alcohol and then the production of another differential metabolite, Syringin, which is catalyzed by the UGT72E enzyme. Coniferin, another differential metabolite enriched in this pathway, was generated from Coniferyl alcohol catalyzed by UGT72E. The trends of the two differential metabolites, Syringin and Coniferin, were similar and both of them were regulated by the UGT72E enzyme. The trend of UGT72E enzyme activity was basically the same as that of the two metabolites, except that the metabolites decreased and the enzyme activity increased during the 16 h drying period.

## 4. Discussion

This present study revealed that rhubarb undergoes significant changes in color parameters (L*, a* and b*) during the drying process, especially in the early stages of drying. This is in line with the findings of Zhou et al. [38], who found that sun-dried Kampot tea showed a significant decrease in L*, a*, and b*, that is, the sun-drying process reduces the brightness, redness, and yellowness of the tea. These changes are indicative of an enzymatic browning process in which PPO catalyzes the oxidation of phenolic compounds to quinones, and the subsequent polymerization of these quinones forms melanin [39]. Notably, PPO activity peaked at 4 h of drying (Figure 3B), coinciding with a significant rise in BI values and the most pronounced color changes. This synchronicity confirms PPO as the key enzyme driving browning [40]. In addition, rhubarb phenol, the most abundant AQ compound in rhubarb, had its highest content at 4 h of drying (Figure 3D), and at the same time the activity of PPO was significantly enhanced. PPO catalyzed the oxidation of polyphenols in rhubarb to form quinone compounds [41]. This is likely to be one of the direct causes of the decrease in AQ content in the subsequent period [42]. In addition, the gradual reduction in water within the plant tissues with increasing drying time resulted in the gradual release of water-bound AQs and their possible degradation or transformation. In particular, free AQs may be more susceptible to decomposition during sun drying due to their relatively poor thermal stability. The biphasic trend of CAT activity (Figure 3A)—initial increase (4–8 h), subsequent decline (8–12 h), and final resurgence (12–16 h)—aligns with plant adaptive responses to oxidative stress. During early drying, CAT scavenges ROS to protect cellular integrity. Its later resurgence likely reflects de novo synthesis to counteract prolonged oxidative damage, as reported in drought-stressed *Arabidopsis* [43]. These findings highlight the complex interplay between enzyme activity and the quality of dried rhubarb, highlighting the need to optimize drying conditions to maintain medicinal properties.

In studying the drying kinetics of rhubarb, the variation in DR was closely related to the thickness of the slices. In particular, the 2 mm slices exhibited faster DR (Figure 4B), which was attributed their shorter moisture migration paths and higher surface area-to-volume ratio under drying conditions [44]. In addition, at the beginning of the drying process, the treatment groups with all four slice thicknesses exhibited a higher DR, which could be attributed to the rapid evaporation of water from the sliced surfaces, and the DR gradually decreased as the DT increased. This finding is in agreement with the results of Yue et al., who found that *Codonopsis pilosula* rapidly absorbed energy and evaporated water during the initial drying period. After reaching the decreasing rate drying period, the surface structure of the material becomes dense due to the decrease in water content, which reduces the rate of water vapor transfer within the material [45]. The logarithmic model provided a better simulation of the rhubarb drying process throughout the drying process. Amer et al. found that the Midili model was the best model to describe the drying kinetics of chamomile [46]. Similarly, Zhang et al. investigated the drying kinetic properties of broccoli by various mathematical models in their study of far-infrared drying of broccoli and found that the modified Page, Page, and Wang and Singh models were better able to fit the drying process of broccoli in the temperature range of 60 °C to 80 °C [47]. This suggests that the selection of an appropriate mathematical model is crucial for describing and predicting the behavior of drying kinetics, which can provide a better understanding of the mechanism of moisture migration during the drying process and provide a theoretical basis for the improvement of rhubarb drying technology.

The observed increase in chalcone isomerase (CHI) activity coupled with the decreased abundance of liquiritigenin and naringenin during the late drying stage in the flavonoid biosynthesis pathway may result from branch competition of metabolic flux and oxidative stress-mediated regulation. Liquiritigenin is converted into garbanzol and butin via F3H and TT7, respectively (Figure S1), while naringenin enters nine downstream branches (e.g., CYP75A-catalyzed eriodictyol synthesis). Additionally, both metabolites serve as precursors for the isoflavonoid biosynthesis pathway, suggesting that elevated CHI activity during late drying may divert precursors toward isoflavonoid production. In salt-stressed soybean (*Glycine max*), the transcription factor GmERD15c upregulates CHS and F3H genes, driving the synthesis of defensive isoflavonoids (e.g., quercetin) while reducing basal flavonoids like naringeni [48]. Similarly, in pigeon pea (*Cajanus cajan*), salt stress enhances isoflavonoid synthesis (e.g., genistein) while suppressing flavanones and flavones [49]. These findings indicate that environmental stresses (e.g., dryin) redistribute metabolic flux by modulating branch enzyme activities, leading to the depletion of core intermediates.

In tyrosine metabolism (Figure S2), the synthesis of the downstream metabolite 3-fumarylpyruvate involves the coordinated action of the nagl, FAHD1, and nagk enzymes. However, the asynchronous trends between enzyme activities and metabolite abundance reveal a competitive substrate allocation mechanism. 3-Maleylpyruvate can be converted to 3-fumarylpyruvate via nagl, followed by FAHD1/nagk-mediated production of pyruvate and fumarate, or it can be directly hydrolyzed to pyruvate by maleylpyruvate hydrolase. Such multi-enzyme competitive regulation is widespread in plant metabolism. For instance, polyphenol synthesis in *Rheum tanguticum* relies on balanced enzyme activities, while α-chaconine biosynthesis in tomato (*Solanum lycopersicum*) requires coordinated multi-enzyme action [50]. Notably, FAHD1 activity fluctuations may correlate with its oxidative modification status. Recent studies demonstrate that plant FAHD1 homologs undergo cysteine thiolation under oxidative stress, leading to conformational changes and activity suppression [51].

In the phenylpropanoid biosynthesis pathway (Figure S3), the synthesis of Sinapic acid is regulated by the coordinated action of multiple enzymes, including OMT1, 4CL1, and ALDH2C4. The decline in 4CL1 activity during the late drying stage may result from feedback inhibition caused by Sinapoyl-CoA accumulation. A similar mechanism has been reported in the phenylpropanoid pathway of *Arabidopsis thaliana*, where excessive accumulation of cinnamic acid derivatives (e.g., sinapate) suppresses 4CL activity to reduce CoA ester synthesis, thereby balancing metabolic flux [52]. Additionally, post-translational modifications (e.g., phosphorylation) may alter 4CL1 activity by modifying its conformation or stability. For example, phosphorylation enhances the activity of the 4CL homolog Os4CL3 in rice (*Oryza sativa*) under salt stress, suggesting a similar regulatory mechanism may operate during rhubarb drying [53]. UGT72E regulates the synthesis of both Syringin and Coniferin, but its activity inversely correlates with metabolite abundance at 16 h of drying. This discrepancy may arise from substrate competition or negative feedback regulation. UGT family enzymes often exhibit substrate promiscuity, and their catalytic efficiency can be dynamically influenced by substrate concentrations [54]. These findings reveal the complex regulatory relationships between metabolites and key enzymes within the biosynthetic pathway during rhubarb drying, providing an important basis for further study and regulation of plant secondary metabolism.

## 5. Conclusions

This study systematically revealed the influence of slice thickness on drying kinetics and bioactive component metabolism during the sun-drying of rhubarb, demonstrating that 2 mm-thick slices achieved optimal synergy between bioactive component retention and drying efficiency, while 4 mm-thick slices attained the best balance between drying efficiency and appearance quality. These findings can be directly translated into industrial operational standards, providing data-driven support for upgrading traditional herb drying processes. In the field of functional food development, by leveraging the enzymatic regulatory mechanisms in the flavonoid biosynthesis pathway during drying a “photo-thermal synergistic enrichment process” could be designed to create high-value products (e.g., rhubarb antioxidant chewable tablets and lipid-lowering herbal tea). Concurrently, a natural preservation strategy with reduced preservatives can be achieved through citric acid-mediated pH-a_w_ synergistic regulation technology. Future research should focus on constructing a cross-scale model integrating “drying process-metabolic regulation-microbial safety” and developing adaptive drying equipment (e.g., thickness-temperature coupled control systems) with Internet of Things technology, thereby facilitating the transition of rhubarb processing from primary processing to precision manufacturing. This work exemplifies the implementation of the “green manufacturing” strategy in traditional Chinese medicine.

## Figures and Tables

**Table 1:** Fitting results of each drying kinetic model.

Model Name	Slice Thickness (mm)	Evaluation Metrics		
		R^2^	RMSE	χ^2^
Lewis	2	0.99082	0.01969	0.00039
	4	0.99087	0.02230	0.00050
	6	0.99345	0.02742	0.00075
	8	0.99504	0.02939	0.00086
Wang and Singh	2	0.92138	0.04897	0.00240
	4	0.92468	0.07239	0.00524
	6	0.93373	0.07854	0.00617
	8	0.97205	0.08925	0.00797
Weibull	2	0.99113	0.01966	0.00039
	4	0.99308	0.02132	0.00045
	6	0.99425	0.02648	0.00070
	8	0.99528	0.02759	0.00076
Logarithmic	2	0.99205	0.02047	0.00042
	4	0.99433	0.02075	0.00043
	6	0.99513	0.02163	0.00047
	8	0.99519	0.02954	0.00087

**Table 2:** Fitting data based on the logarithmic model.

Slice Thickness (mm)	Model Parameters			Evaluation Indicators		
	a	k	c	R^2^	RMSE	χ^2^
2	1.03643	0.18951	−0.00856	0.99205	0.02954	0.00087
4	0.99628	0.10455	−0.00406	0.99513	0.02047	0.00042
6	0.98489	0.08023	−0.01325	0.99433	0.02163	0.00047
8	1.03497	0.05724	−0.06299	0.99519	0.02075	0.00043

## Data Availability

Data are contained within the article and Supplementary Materials.

## Supplementary Materials

The following supporting information can be downloaded at: https://www.mdpi.com/article/10.3390/biology14080963/s1, Figure S1: KEEG metabolic pathway of Flavonoid biosynthesis, Figure S2: KEEG metabolic pathway of Tyrosine metabolism, Figure S3: KEEG metabolic pathway of Phenylpropanoid biosynthesis; Table S1: Mathematical expressions for the drying kinetics model, Table S2: Significance analysis of enzyme activities, Table S3: Significance analysis of relative abundance of differential metabolites.
