# Supplementary material for: Mechanistic Elucidation and Establishment of Drying Kinetic Models of Differential Metabolite Regulation in Rheum palmatum During Natural Sun Drying: An Integrated Physiology, Untargeted Metabolomics, and Enzymology Study
Source: Biology (Basel). 2025 Aug 1;14(8):963. doi: 10.3390/biology14080963 (PMC12383960; doi:10.3390/biology14080963)
Supplement: Supplementary file 1 [file biology-14-00963-s001.zip › biology-3591593-supplementary.pdf]

Table S1 Mathematical expressions for the drying kinetics model

Model number	Model name	Model expression
1	Lewis	MR=exp（-kt）
2	Wang and singh	$MR=1+at+bt^2$
3	Weibull	$MR=\exp[-(t/\alpha)^{\beta}]$
4	Logarithmic	MR=a exp（-kt）+c

MR is the moisture ratio under different drying methods; a、b、c、k、α、β are coefficients; t is the drying time (h)

Table S2 Significance analysis of enzyme activities

Name	Time(h)				
	4	6	8	12	16
CHI	1075.00 ± 47.46^{ab}	1217.22 ± 101.67^{a}	746.67 ± 38.44^{c}	858.33 ± 129.79^{c}	1032.78 ± 110.46^{b}
CYP75A	33.25 ± 2.84^{c}	53.69 ± 2.94^{b}	50.42 ± 3.21^{b}	73.14 ± 2.82^{a}	35.06 ± 3.92^{c}
TT7	94.55 ± 1.72^{c}	106.94 ± 1.78^{b}	104.95 ± 1.94^{b}	118.72 ± 1.71^{a}	95.64 ± 2.38^{c}
F3H	94.55 ± 1.92^{a}	96.89 ± 0.88^{a}	81.30 ± 1.53^{b}	81.80 ± 1.17^{b}	80.52 ± 1.81^{b}
ANR	544.58 ± 27.42^{a}	487.08 ± 9.15^{b}	514.79 ± 7.71^{ab}	494.38 ± 16.67^{b}	384.17 ± 26.26^{c}
PDS1	34.64 ± 2.40^{ab}	30.69 ± 2.63^{bc}	33.71 ± 3.76^{ab}	37.55 ± 3.28^{a}	26.46 ± 1.5^{c}
HGO	82.65 ± 2.11^{a}	75.93 ± 2.34^{b}	77.34 ± 1.22^{b}	81.84 ± 0.92^{a}	80.87 ± 1.49^{a}
nagL	58.67 ± 3.70^{ab}	55.59 ± 2.25^{b}	62.66 ± 1.05^{a}	54.17 ± 2.64^{b}	46.98 ± 2.06^{c}
FAHD1	93.38 ± 5.65^{b}	89.84 ± 4.05^{b}	104.20 ± 2.26^{a}	91.78 ± 1.82^{b}	88.36 ± 1.55^{b}
nagK	130.61 ± 4.27^{c}	141.95 ± 10.14^{abc}	148.41 ± 5.67^{a}	144.67 ± 5.04^{ab}	134.76 ± 2.80^{bc}
OMT1	2943.33 ± 60.02^{a}	2657.78 ± 95.02^{b}	2911.67 ± 39.83^{a}	2981.67 ± 126.57^{a}	2992.78 ± 96.27^{a}
UGT84A2	179.91 ± 8.88^{a}	139.72 ± 20.93^{b}	189.44 ± 16.02^{a}	180.65 ± 10.19^{a}	169.81 ± 7.90^{a}
ALDH2C4	26.38 ± 3.09^{b}	31.63 ± 2.29^{a}	29.56 ± 0.66^{ab}	29.04 ± 1.12^{ab}	16.47 ± 0.76^{c}
4CL1	45.18 ± 1.09^{c}	51.31 ± 1.92^{a}	48.23 ± 1.88^{b}	48.43 ± 1.23^{ab}	50.11 ± 1.27^{ab}
UGT72E	13.41 ± 0.97^{b}	$11.08\pm0.96b^{c}$	2.97 ± 0.67^{d}	9.60 ± 1.75^{c}	25.08 ± 3.09^{a}

Note:chalcone isomerase (CHI), anthocyanidin reductase (ANR), flavonoid 3'-monooxygenase (TT7), flavanone 3-hydroxylase (F3H), flavanoid 3',5'-hydroxylase (CYP75A), 4-

hydroxyphenylpyruvate dioxygenase (PDS1), maleylpyruvate isomerase (nagL), homogentisate 1,2-dioxygenase (HGO), acylpyruvate hydrolase (FAHD1), 3-fumarylpyruvate hydrolase (nagK), caffeate O-methyltransferase (OMT1), coniferyl-aldehyde dehydrogenase (ALDH2C4), coniferyl-alcohol glucosyltransferase (UGT72E), sinapate 1-glucosyltransferase (UGT84A2), and 4-coumarate—CoA ligase (4CL1)

Table S3 Significance analysis of relative abundance of differential metabolites

Name	Time(h)				
	4	6	8	12	16
Liquiritigenin	158578441.38±6034873.58^{b}	315502177.87±7097287.20^{a}	46181200.98±4380253.03^{d}	137284609.48±8942572.25^{c}	43157285.16±21159033.16^{d}
Naringenin	52951842.46±2276248.18^{b}	108563905.01±32305817.85^{a}	24438258.55±1215584.59^{cd}	39243693.09±11399014.16^{bc}	13907199.73±567760.66^{d}
(-)-Epicatechin	833560357.97±62025664.59^{a}	782573768.62±34833507.33^{a}	797515998.20±71189867.00^{a}	707558195.92±53265770.40^{b}	501862822.48±57306193.86^{c}
Homogentisate	9220825.72±1175698.87^{a}	5456716.44±1310816.19b^{c}	6415018.85±164748.12^{b}	6646852.54±1477295.74^{b}	4213439.30±881757.27^{c}
3-Fumarylpyruvate	4038237.93±193659.85^{c}	4652759.41±432649.26^{bc}	12149640.74±985339.34^{a}	5504165.78±462419.86^{b}	11991461.98±1273160.19^{a}
Sinapic acid	11064480.63±1022763.44$^{\mathbf{b}}$	12684077.95±854987.31^{a}	3613526.01±350937.03^{d}	9614067.81±707617.29^{c}	3883654.19±255841.15^{d}
Coniferin	80062328.14±2623707.53^{a}	69265697.80±2911749.53^{b}	4255562.08±372780.56^{d}	58983299.31±3310216.15^{c}	2828969.75±218371.81^{d}
Syringin	170743644.77±6432502.38^{a}	153569189.12±5659210.28^{b}	24719408.68±4739625.64^{d}	136900115.30±8174204.68^{c}	26371511.17±791678.59^{d}

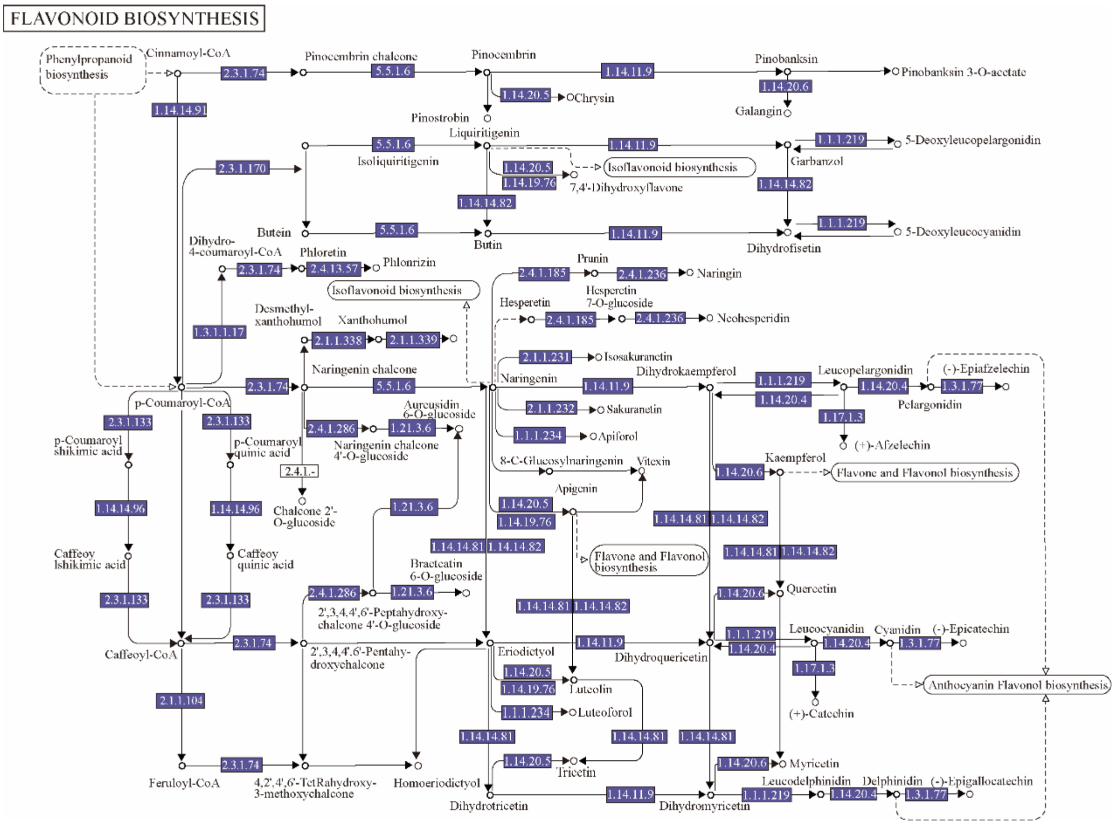

Figure S1 KEEG metabolic pathway of Flavonoid biosynthesis

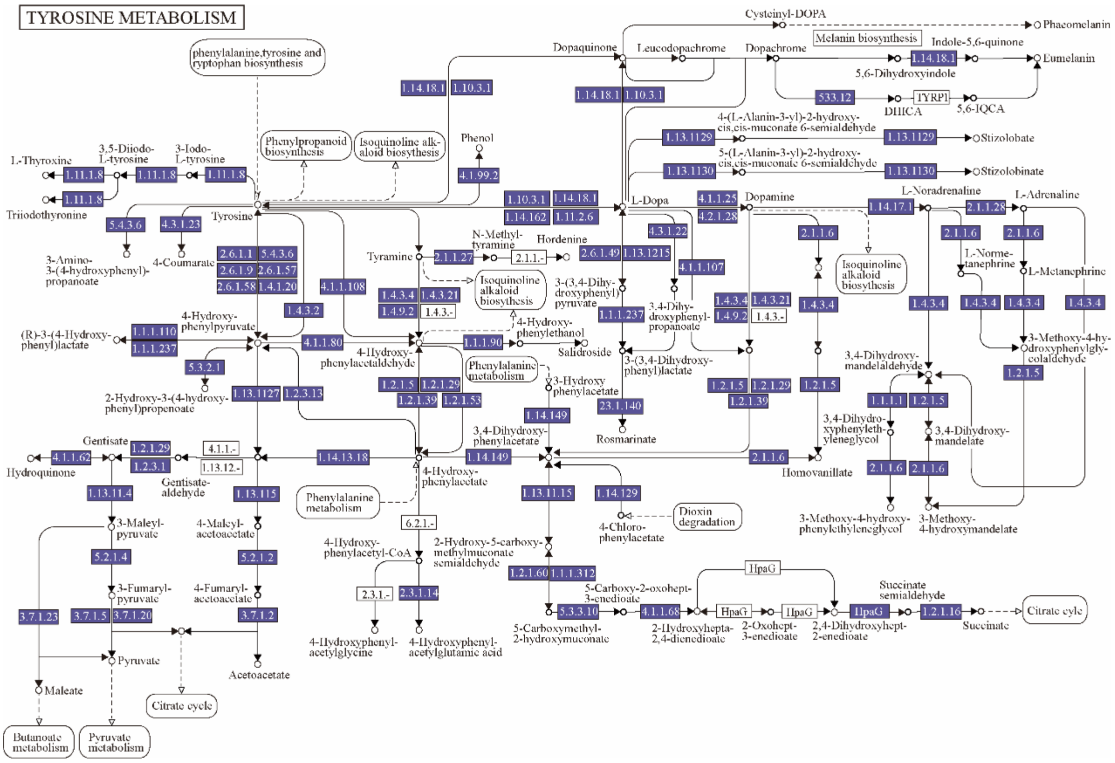

Figure S2 KEEG metabolic pathway of Tyrosine metabolism

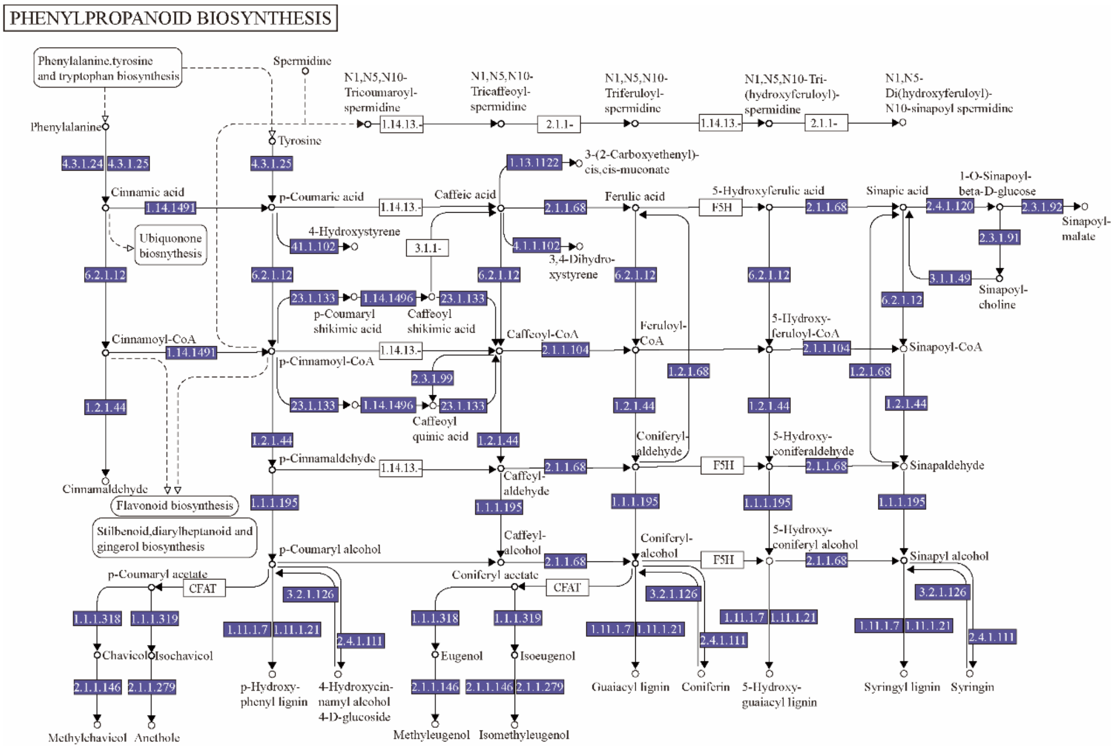

Figure S3 KEEG metabolic pathway of Phenylpropanoid biosynthesis
